# Dental implant as a potential risk factor for maxillary sinus fungus ball

**DOI:** 10.1038/s41598-024-52661-9

**Published:** 2024-01-30

**Authors:** Sun A. Han, Sungtae Kim, Yuju Seo, Seung Koo Yang, Chae-Seo Rhee, Doo Hee Han

**Affiliations:** 1 Department of Otorhinolaryngology-Head and Neck Surgery, Hallym University Dongtan Sacred Heart Hospital, Hwaseong-si, Republic of Korea; 2 Department of Periodontology, Dental Research Institute, Seoul National University School of Dentistry, Seoul, Republic of Korea; 3 Department of Otorhinolaryngology-Head and Neck Surgery, Seoul National University College of Medicine, Daehakro 101, Jongno-gu, Seoul, Republic of Korea; 4 Graduate School of Immunology, Seoul National University College of Medicine, Seoul, Republic of Korea; 5 Institute of Allergy and Clinical Immunology and Sensory Organ Research Institute, Seoul National University Biomedical Research Center, Seoul, Republic of Korea; 6 Sensory Organ Research Institute, Seoul National University Biomedical Research Center, Seoul, Republic of Korea

**Keywords:** Dental diseases, Respiratory tract diseases, Dentistry, Infection, Risk factors

## Abstract

Fungus ball is the most common form of non-invasive fungal sinusitis, and maxillary sinus is the most commonly involved site. Maxillary sinus fungus ball (MFB) accounts for a considerable proportion of unilateral maxillary sinusitis. The prevalence of MFB has recently increased; however, its contributing factors are unclear. This study analyzed the association between MFB and dental implants. One hundred one patients who underwent unilateral maxillary sinus surgery were divided into two groups based on surgical biopsy results: unilateral bacterial sinusitis (UBS, n = 45) and MFB (n = 56). Stratified random sampling of 30 patients from each group was performed to adjust for age. The number of dental implants on maxillary teeth and degree of penetration into the maxillary sinus was radiologically evaluated. The number of patients with dental implants was greater (*P* = 0.085) and the number of implants was significantly higher (*P* = 0.031) in the MFB group. Dental implant can be a potential risk factor for MFB development. Therefore, dental implant surgeons should take caution in penetrating the maxillary sinus floor during implant insertion and otolaryngologists should consider the possibility of fungus ball when assessing patients with sinusitis who have dental implants.

## Introduction

Fungus ball is the most common form of non-invasive fungal sinusitis. It is characterized by accumulation of fungal debris in the sinus without mucosal invasion. Maxillary sinus is the most common site of fungus ball formation in the nasal cavity and among paranasal sinuses. Previous studies, including a large multicenter study, have reported increasing prevalence of sinonasal fungus ball^1,2^. Distinguishing between bacterial sinusitis and maxillary sinus fungus ball (MFB) is important because they require different treatments. Odontogenic bacterial sinusitis is initially treated with antibiotics and management of the infected teeth, whereas symptomatic fungus ball requires surgical removal. Several studies have focused on identifying fungus balls on preoperative imaging^3,4^. In the globally ageing society, increased number of elderly have dental problems and dental implant is one of the main methods of oral rehabilitation^5^.

While other dental procedures, such as endodontic treatment, have been suggested as risk factors for MFB formation^6–8^, the causative relationship between dental implant and MFB has not been established. In contrast to previous investigations, the current study analyzed the number of dental implants and their degree of penetration into the maxillary sinus. In this study, we aimed to investigate whether dental implant is a potential risk factor for the development of MFB.

## Results

### Patient characteristics

**Figure 1:**
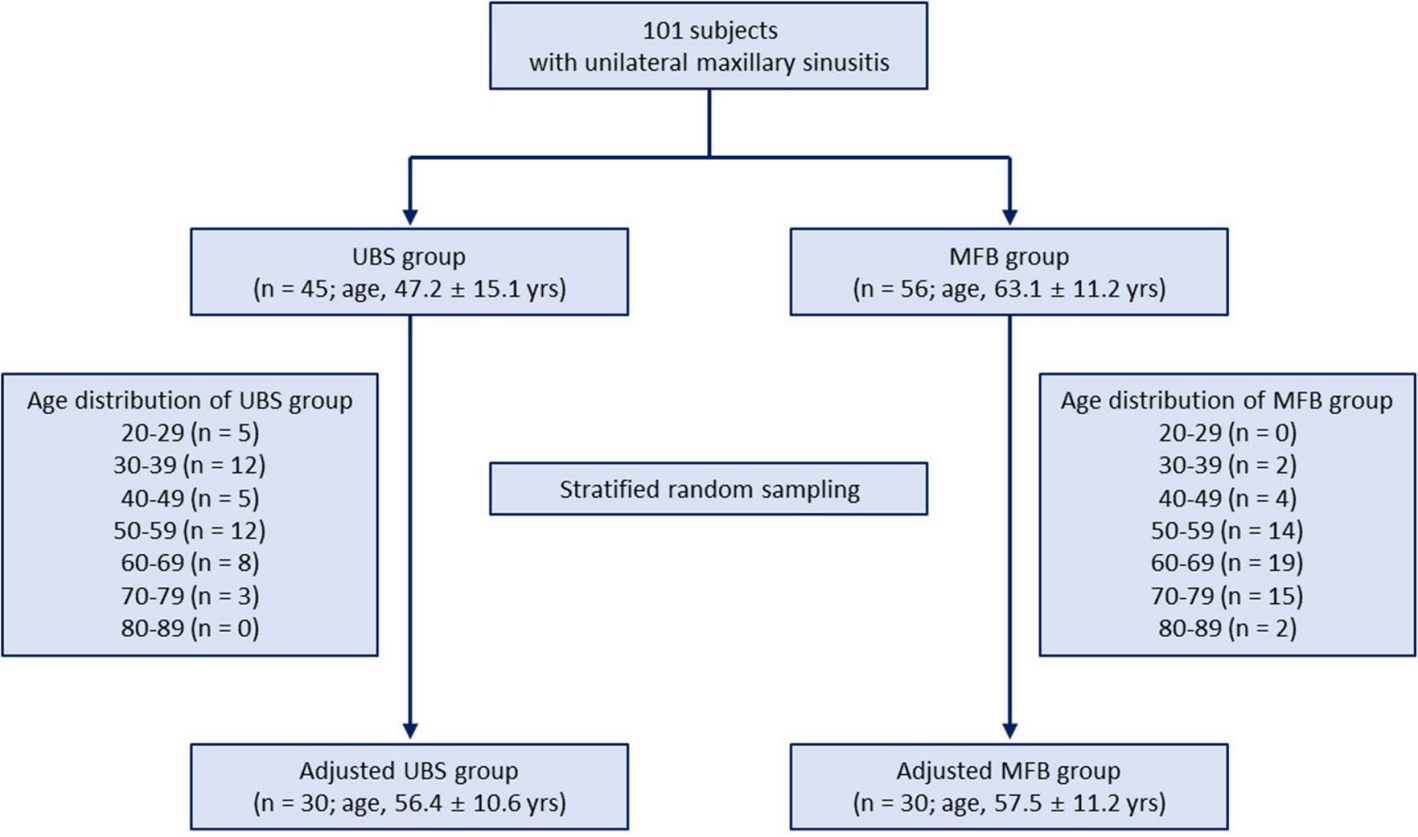
Flow chart of patient selection. UBS, unilateral bacterial sinusitis; MFB, maxillary sinus fungus ball.

Of the 101 patients analyzed, 45 patients (44.6%) had UBS and 56 patients (55.4%) had MFB. No significant difference was found in the male-to-female ratio between the UBS and MFB groups (20:25 vs. 19:37, *P* = 0.309); however, the mean age of the MFB group was significantly higher than that of the UBS group (63.1 ± 11.2 years vs. 47.2 ± 15.1 years, ***P < 0.001). Demographics of the patients analyzed are shown in Table 1. To adjust for age as a confounding variable, stratified randomization was performed after classifying patients into each decade (Fig. 1). Sixty age-matched patients—30 patients in each group—were selected. In the age-matched groups, the mean age was not different between the groups (UBS: 56.4 ± 10.6 years vs. MFB: 57.5 ± 11.2 years). In the age-matched UBS group, five patients had diabetes mellitus and one patient had liver cirrhosis, and no patient had hematological malignancy, was taking immunosuppressants, or had undergone chemotherapy. In the age-matched MFB group, five patients had diabetes mellitus, two had liver cirrhosis, and one was taking immunosuppressants after kidney transplant, and no patient had hematological malignancy or had undergone chemotherapy. No statistically significant difference was found between the two groups (Supplementary Table S1).

**Table 1:** Demographic characteristics of patients with unilateral maxillary sinusitis.

Unadjusted data			
	UBS (n = 45)	MFB (n = 56)	*P* value
Age, years	47.2 ± 15.1	63.1 ± 11.2	< 0.001
Age group, n (%)			0.001
< 65 years	38 (84.4)	29 (51.8)	
≥ 65 years	7 (15.6)	27 (48.2)	
Sex, n (%)			0.309
Male	20 (44.4)	19 (33.9)	
Female	25 (55.6)	37 (66.1)	

Age-adjusted data			
	UBS (n = 30)	MFB (n = 30)	*P* value
Age, years	56.4 ± 10.6	57.5 ± 11.2	0.682
Age group, n (%)			
< 65 years	22 (73.3)	22 (73.3)	
≥ 65 years	8 (26.7)	8 (26.7)	
Sex, n (%)			
Male	12 (40.0)	11 (36.7)	
Female	18 (60.0)	19 (63.3)	

*UBS* unilateral bacterial sinusitis, *MFB* maxillary sinus fungus ball.

### Dental implants in the UBS and MFB groups

Ten of the 101 patients analyzed had dental implants. Eight of them were male, and their average age was 63.10 ± 11.0 years. One patient (2.2%) in the UBS group and nine patients (16.1%) in the MFB group underwent placement of dental implants (*P = 0.021; OR, 8.43, 95% CI, 1.03–69.24).

In age-adjusted data (n = 60), the number of patients with pathological maxillary teeth on the same side of maxillary sinusitis was 21 (70.0%) in the UBS group and 19 (63.3%) in the MFB group (Supplementary Table S2). The number of patients with dental implants was higher in the MFB group than in the UBS group [1/30 (3.3%) vs. 5/30 (16.7%), *P* = 0.085], but statistical significance was not achieved (Table 2). Comparison of the total number of pathological maxillary teeth showed that the number of dental implants was significantly higher in the MFB group than in the UBS group [2/120 (1.7%) vs. 9/120 (7.5%), *P = 0.031]. Additional analysis was performed only on the odontogenic sinusitis cases, excluding those without dental problems on the CT scan. Even when only odontogenic cases were examined, the incidence of dental implants was higher in the MFB group than in the UBS group (Table 2). Data on the number of patients and number of pathological teeth other than implants are shown in Supplementary Table S2.

**Table 2:** Number of patients and number of pathological teeth in the UBS and MFB groups.

	Number of affected patients, n/total n (%)				Number of affected teeth, n/total maxillary teeth n (%)			
	UBS	MFB	*P* value	OR (95% CI)	UBS	MFB	*P* value	OR (95% CI)
Pathological maxillary teeth	21/30 (70)	19/30 (63.3)	0.583	0.74 (0.25–2.17)	39/120 (32.5)	37/120 (30.8)	0.781	0.93 (0.54–1.60)
Dental implant	1/30 (3.3)	5/30 (16.7)	0.085	5.80 (0.63–53.01)	2/120 (1.7)	9/120 (7.5)	0.031	4.78 (1.01–22.63)
Dental implant in odontogenic sinusitis	1/21 (4.8)	5/19 (26.3)	0.057	7.14 (0.75–67.97)	2/84 (2.4)	9/76 (11.8)	0.018	5.51 (1.15–26.36)

*UBS* unilateral bacterial sinusitis, *MFB* maxillary sinus fungus ball.

### Degree of dental implant penetration into the maxillary sinus and their distribution

Half of the implants in the UBS group (1/2) and 11.1% (1/9) of the implants in the MFB group were classified as grade 1. Half of the implants in the UBS group (1/2) and 33.3% (3/9) of the implants in the MFB group were classified as grade 2. Approximately 55.6% (5/9) of the implants in the MFB group were classified as grade 3, whereas no implants in the UBS group were classified as grade 3. Statistical analysis showed no significance in the linear-by-linear association (*P* = 0.124). The UBS group had no dental implants in the premolar region but had two implants in the molar region. In the MFB group, two implants were located in the premolar region, and seven implants in the molar region.

## Discussion

In this study, we investigated the association between dental implants and development of MFB. We found that the number of patients with dental implants was higher in the MFB group than in the UBS group; however, statistical significance was not achieved. Further, comparison of the number of teeth showed that the number of dental implants was significantly higher in the MFB group than in the UBS group. All of the dental implants with grade 3 penetration were those of the MFB group while dental implants in the UBS group were all classified into grade 1 or 2.

Cases of fungus ball arising in the background of dental implants, including zygomatic implants have been reported^9–12^. In a previous study that examined odontogenic factors in MFB compared with the healthy side, the overall presence of odontogenic factors was correlated with fungus balls, whereas particular factors, such as the presence of dental implants, were not significantly related^13^. However, this study included only four patients with dental implants in each group. Furthermore, the large difference in the number of patients with tooth extraction (n = 145) and dental implants (n = 8) suggests that tooth extraction does not ensue dental implant in other countries probably owing to medical costs or socioeconomic factors. With increasing number of elderly in the globally aging population, more patients complain of dental problems. As dental implant is one of the main methods of oral rehabilitation, the dental implant market is steadily increasing. In Korea, dental implant- placement is a common dental procedure, and an increasing number of patients are considering this procedure particularly after its coverage by the Korean National Health Insurance. This is possibly reflected by the greater prevalence of dental implants in our study.

**Figure 2:**
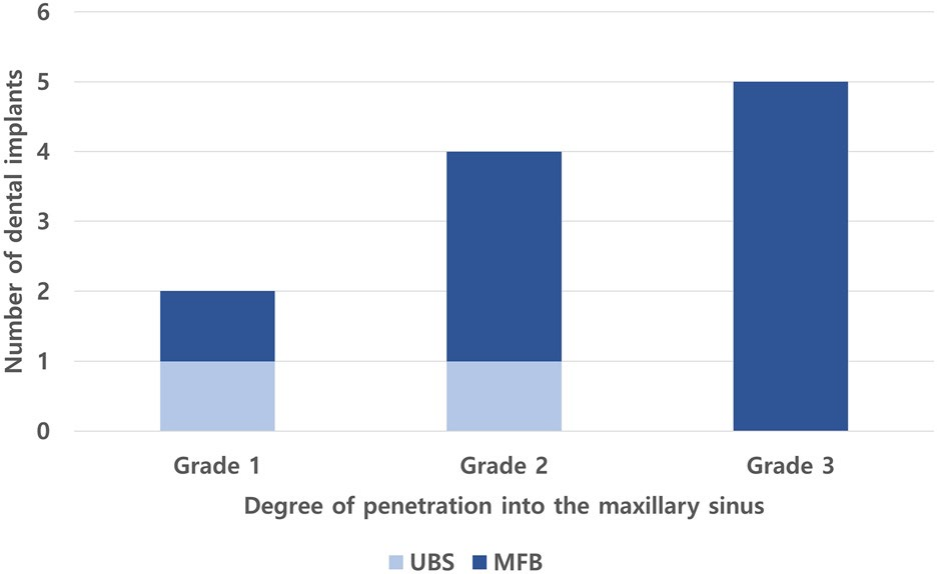
The number of implants according to degree of penetration into the maxillary sinus. *UBS* unilateral bacterial sinusitis, *MFB* maxillary sinus fungus ball.

Biomaterial-associated infection is a major complication of dental implants and accounts for approximately 14% of total implant failures^14^, and it is usually mediated by biofilm formation^15^. Biofilms are formed when microbes adhere to the surface of a medical device and proliferate, resulting in several layers of microorganism clusters. These microorganism clusters detach from the macrocolony, leading to a spread of the infection^16^. Bacterial biofilms have been suggested to play a role in the pathogenesis of chronic rhinosinusitis^17^. In in vitro models, including that of human sinonasal epithelium, *Aspergillus* was proven to form biofilm structures^15,18^. Similarly, a study investigated the formation of fungal biofilm by *Candida albicans* on dental implants^19^. Given that dental implant surface can act as a substrate for biofilm formation^20^, implants provide a potential medium for fungus ball development. We evaluated the images focusing on the premolar and molar teeth in proximity to the maxillary sinus. Maxillary teeth consist of two premolar and two molar teeth, and the proximity between the roots of the maxillary teeth and maxillary sinus is a challenge in dentistry, particularly during dental implant placement, because of the risk of penetration into the maxillary sinus. A study has shown that sinus membrane perforation did not affect implant survival and sinus membrane thickening^21^, and from our experience not all implants penetrating the maxillary sinus floor on preoperative CT scans show exposed implants during surgery as shown in Supplementary Fig. 1. However, in extreme cases, dental implants can be displaced into the maxillary sinus requiring surgical treatment^22^, and shorter implants have been used as alternatives to overcome having to perform maxillary sinus lifts^23^. We also analyzed the distribution of dental implants in the premolar and molar regions of each group. In the MFB group, the molar region had three times more implants than the premolar region. The distance between the molar and maxillary sinus is closer than that between the premolar and maxillary sinus^24^. Furthermore, the incidence of MFB was higher with increasing degree of implant penetration (Fig. 2). Based on these results, we hypothesized that the direct contact between the dental implant and *Aspergillus* contributes to MFB development. We suggest three grounds on which we based this hypothesis.

First, inhalation of fungal elements is unavoidable during normal respiration; fungus is routinely deposited within the nose and paranasal sinuses^25^. Fungal spores introduced into the maxillary sinus have a higher chance of contacting the implant when the implant is exposed to the maxillary sinus, potentially serving as a medium for biofilm formation on the implant surface. This could possibly explain the positive correlation between the degree of implant penetration and the incidence of MFB (Fig. 2).

Second, the environment of the inflamed maxillary sinus is possibly more vulnerable to fungal compared with bacterial biofilm formation. When the sinus ostium is obstructed, oxygen tension is reduced, creating an anaerobic environment. Aerobes, such as *Staphylococcus aureus*, *Staphylococcus epidermidis*, *Streptococcus viridans*, and *Pseudomonas aeruginosa*, are the main microorganisms responsible for biofilm formation on indwelling medical devices^26^; however, *Aspergillus* survives in an anaerobic environment and even in low-level nutritional environment^27^.

Finally, implant properties and implant status may affect development of fungus balls. Studies have shown that peri-implantitis in sinus floor augmentation may be related to maxillary sinusitis^28^ and periodontitis is associated with increased risks of fungus balls (adjusted hazard ratio, 1.46; p = 0.002)^29^. Rough-surfaced implants promote osseointegration; however, the rough surfaces increase the risk of peri-implantitis^30^. Various approaches, including antibacterial surfaces and coatings that release antibiotics such as vancomycin and gentamycin, have been applied to prevent peri-implantitis^14^. These modifications on implant surface are mostly focused on antibacterial, rather than antifungal effects. The development and use of antifungal implant materials and early treatment of peri-implantitis may subsequently reduce the prevalence of fungal infections.

Although there have been some case reports, the relationship of fungus ball with dental implant is not well established. Several studies have reported the relationship between root canal treatment and other dental procedures with fungus ball; however, this is the first study to focus on the relationship with dental implants. Furthermore, we have analyzed the dental implant’s degree of penetration into the maxillary sinus.

This study has some limitations. First, it was cross-sectional in nature; thus, we were not able to assess the time interval between the dental procedure and the occurrence of MFB. This indicates the possibility that the patients who underwent root canal treatment or dental implant placement without MFB at the time of CT scan may develop MFB in the future. Second, because this was a retrospective cohort study, we could not obtain information on the status of the patients’ implants such as peri-implantitis. Characteristics of each implant such as surface properties and design or the presence of biofilm on the dental implants could not be assessed in this study. Finally, the limited sample size at tertiary referral hospital may preclude generalization of the findings to other populations. Further prospective studies with larger sample size assessing the status of the implant are warranted to allow a more detailed, highly powered statistical evaluation of dental procedure-related factors.

In conclusion, dental implant can be a potential risk factor for MFB development. With increasing dental implant cases, dental implant surgeons should take caution in penetrating the maxillary sinus floor during implant insertion. Otolaryngologists should consider the possibility of MFB when assessing patients with unilateral sinusitis who have dental implants.

## Materials and methods

This retrospective study was conducted with the approval of the Institutional Review Board (IRB) of Seoul National University Hospital (SNUH) (IRB approval number: H-1705-125-855) and performed in accordance with Declaration of Hesinki. Informed consent was waived by IRB of SNUH due to the retrospective nature of the study. Patients aged 19 years and older who underwent endoscopic sinus surgery for unilateral maxillary sinusitis at SNUH between January 2016 and February 2017 were included. Those who were treated for postoperative cheek cysts, mucoceles, foreign body-induced sinusitis, and benign or malignant tumors were excluded. A total of 101 patients were included in the final analysis. They were divided into two groups according to the surgical biopsy results: unilateral bacterial sinusitis (UBS) and MFB. Patients with surgical pathology results showing chronic inflammation on hematoxylin and eosin (H&E) staining were determined as having UBS. Those with numerous degenerated fungal hyphae on H&E staining and with fungal organisms that were morphologically consistent with Aspergillosis on Gomori methenamine silver and periodic acid-Schiff staining were categorized into the MFB group. To adjust for age as a confounding factor, 30 age-matched patients were selected from each group using stratified random sampling (Fig. 1). We also evaluated known risk factors for fungal infections, such as diabetes, liver cirrhosis, hematological malignancy, use of medical immunosuppressants, and chemotherapy.

**Figure 3:**
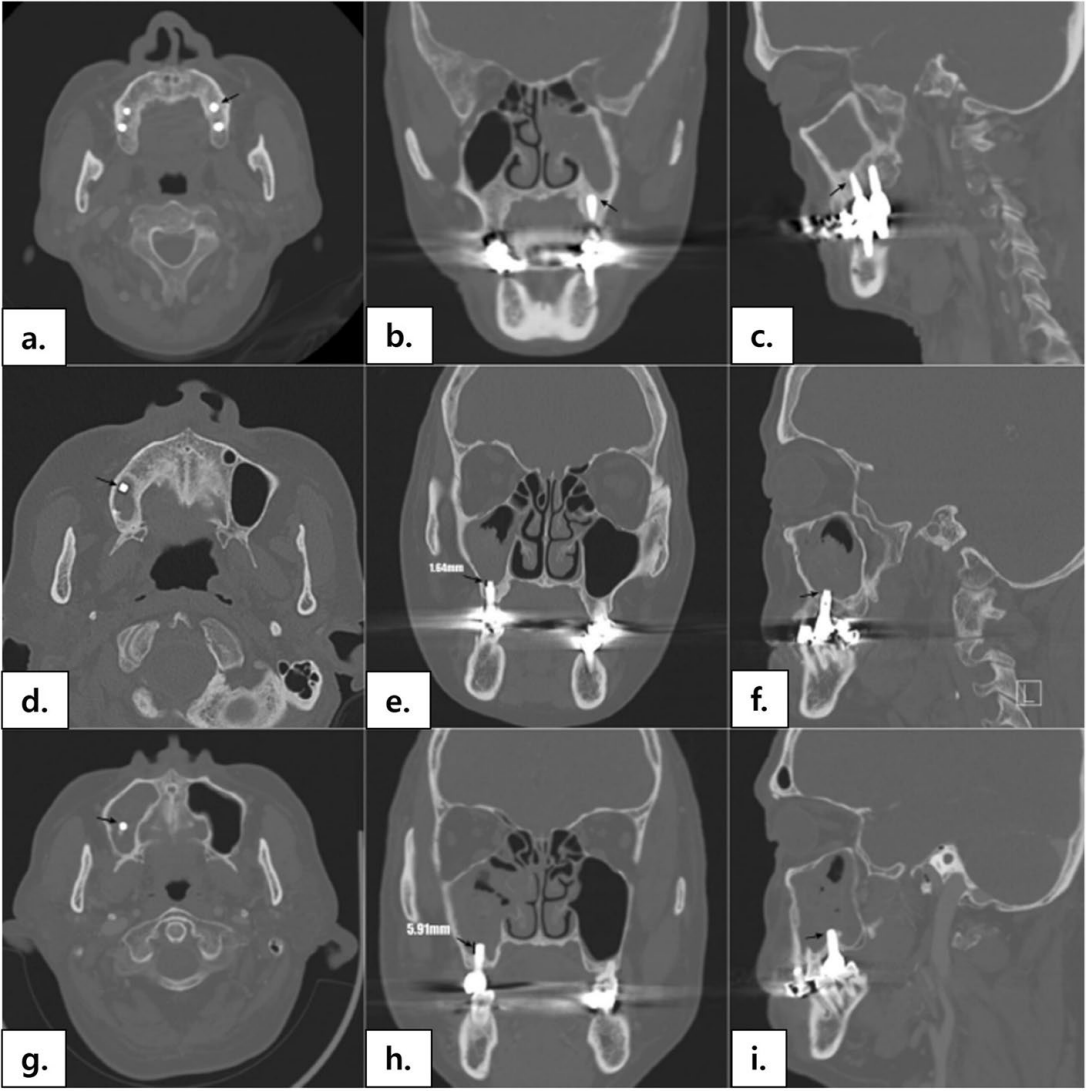
Evaluation of dental implants on paranasal computed tomography: screw part of dental implants are seen as high density materials, some of which penetrates the maxillary sinus floor (black arrows). Grade 1 (**a–c**): dental implant does not penetrate the maxillary sinus floor; Grade 2 (**d–f**): the implant penetrates the maxillary sinus floor but does not 3 mm; Grade 3 (**g–i**): the length of penetration into the maxillary sinus exceeds 3 mm.

Paranasal sinus computed tomography (CT) examination was performed using a helical CT scanner (Genesis Highspeed; GE Medical Systems, Milwaukee, WI) or a multidetector CT scanner (Somatom Sensation 16; Siemens Medical Systems, Erlangen, Germany) for preoperative evaluation. Images were obtained in axial planes and reconstructed into coronal and sagittal planes with a 1–2-mm slice thickness. Two otolaryngologists and one dentist analyzed the CT images for each patient in consensus. The presence of radiographically identifiable dental procedures (root canal treatment and dental implant) and potential sources of odontogenic sinusitis (periapical abscess, oroantral fistula, and a tooth extraction socket) were reviewed. Implants were further classified into three grades according to the degree of penetration into the maxillary sinus (Fig. 3). In grade 1, the implants (Fig. 3a–c) did not penetrate through the bony floor of the maxillary sinus. In grade 2 (Fig. 3d–f), the implants penetrated through the bony floor of the maxillary sinus, but the exposed length did not exceed 3 mm, suggesting intact maxillary sinus floor mucosa. In grade 3 (Fig. 3g–i), the penetrated length of dental implants exceeded 3 mm, suggesting injury of the maxillary sinus floor mucosa. The cutoff value was set at 3 mm because it is the mean thickness of healthy sinus mucosa^31^.

### Statistical analysis

Analyses were performed using SPSS 23.0 software (IBM, Armonk, NY), and statistical significance was set at *P* < 0.05. For sex, number of patients and teeth, and distribution of dental implants in the premolar and molar regions, the Chi-square test or Fischer’s exact test was performed, and odds ratio (OR) and 95% confidence interval (CI) were calculated. For the degree of implant penetration into the maxillary sinus, the linear-by-linear association was performed. The independent t-test was used to compare the differences in age between the UBS and MFB groups. To adjust for age as a confounding factor, age-stratified randomization was performed in Excel’s random number generator function.

### Supplementary Information

Supplementary Figure 1.

Supplementary Tables.

## Data Availability

Data are available from the corresponding author upon reasonable request.

## References

[1] Kim DW (2020). Clinicopathologic characteristics of paranasal sinus fungus ball: Retrospective, multicenter study in Korea. Eur. Arch. Otorhinolaryngol..

[2] Liu X (2020). A retrospective analysis of 1,717 paranasal sinus fungus ball cases from 2008 to 2017. Laryngoscope.

[3] Cha H (2020). Clinical characteristics other than intralesional hyperdensity may increase the preoperative diagnostic accuracy of maxillary sinus fungal ball. Clin. Exp. Otorhinolaryngol..

[4] Seo YJ (2011). Radiologic characteristics of sinonasal fungus ball: an analysis of 119 cases. Acta Radiol..

[5] Kim WJ, Cho YD, Ku Y, Ryoo HM (2022). The worldwide patent landscape of dental implant technology. Biomater. Res..

[6] Mensi M (2007). Risk of maxillary fungus ball in patients with endodontic treatment on maxillary teeth: A case–control study. Oral Surg. Oral Med. Oral. Pathol. Oral Radiol. Endod..

[7] Park GY, Kim HY, Min JY, Dhong HJ, Chung SK (2010). Endodontic treatment: A significant risk factor for the development of maxillary fungal ball. Clin. Exp. Otorhinolaryngol..

[8] Willinger B, Beck-Mannagetta J, Hirschl AM, Makristathis A, Rotter ML (1996). Influence of zinc oxide on Aspergillus species: a possible cause of local, non-invasive aspergillosis of the maxillary sinus. Mycoses.

[9] Lee DH, Yoon TM, Lee JK, Lim SC (2019). Dental implant and fungus ball in the ethmoid sinus. Int. J. Oral Maxillofac. Surg..

[10] Lee JH, Kim JM, Jeong HM, Lee SH (2013). A case of fungal ball accompanied with a microplate as metallic foreign body in maxillary sinus. Korean J. Otorhinolaryngol.-Head Neck Surg..

[11] Sato FR (2010). Aspergillosis of the maxillary sinus associated with a zygomatic implant. J. Am. Dent. Assoc..

[12] Sohn DS, Lee JK, Shin HI, Choi BJ, An KM (2009). Fungal infection as a complication of sinus bone grafting and implants: A case report. Oral Surg. Oral Med. Oral Pathol. Oral Radiol. Endod..

[13] Tomazic PV (2016). Potential correlations of dentogenic factors to the development of clinically verified fungus balls: A retrospective computed tomography-based analysis. Laryngoscope.

[14] Norowski PA, Bumgardner JD (2009). Biomaterial and antibiotic strategies for peri-implantitis: A review. J. Biomed. Mater. Res. B Appl. Biomater..

[15] Beauvais A, Latge JP (2015). Aspergillus biofilm in vitro and in vivo. Microbiol. Spectr..

[16] Veerachamy S, Yarlagadda T, Manivasagam G, Yarlagadda PK (2014). Bacterial adherence and biofilm formation on medical implants: A review. Proc. Inst. Mech. Eng. H.

[17] Cohen M (2009). Biofilms in chronic rhinosinusitis: A review. Am. J. Rhinol. Allergy.

[18] Singhal D, Baker L, Wormald PJ, Tan L (2011). *Aspergillus fumigatus* biofilm on primary human sinonasal epithelial culture. Am. J. Rhinol. Allergy.

[19] Burgers R (2010). Adhesion of *Candida albicans* to various dental implant surfaces and the influence of salivary pellicle proteins. Acta Biomater..

[20] Busscher HJ, Rinastiti M, Siswomihardjo W, van der Mei HC (2010). Biofilm formation on dental restorative and implant materials. J. Dent. Res..

[21] Park WB (2021). Long-term effects of sinus membrane perforation on dental implants placed with transcrestal sinus floor elevation: A case–control study. Clin. Implant. Dent. Relat. Res..

[22] Seigneur M (2023). Characteristics and management of dental implants displaced into the maxillary sinus: A systematic review. Int. J. Oral Maxillofac. Surg..

[23] Carosi P (2021). Short implants (≤ 6mm) as an alternative treatment option to maxillary sinus lift. Int. J. Oral Maxillofac. Surg..

[24] Kosumarl W, Patanaporn V, Jotikasthira D, Janhom A (2017). Distances from the root apices of posterior teeth to the maxillary sinus and mandibular canal in patients with skeletal open bite: A cone-beam computed tomography study. Imaging Sci. Dent..

[25] Soler ZM, Schlosser RJ (2012). The role of fungi in diseases of the nose and sinuses. Am. J. Rhinol. Allergy.

[26] Davey ME, O’Toole GA (2000). Microbial biofilms: From ecology to molecular genetics. Microbiol. Mol. Biol. Rev..

[27] Hall LA, Denning DW (1994). Oxygen requirements of *Aspergillus* species. J. Med. Microbiol..

[28] Park WB, Han JY, Oh SL (2019). Maxillary sinusitis associated with peri-implantitis at sinus floor augmented sites: Case series. Implant Dent..

[29] Kim MG (2023). Periodontitis is associated with the development of fungal sinusitis: A nationwide 12-year follow-up study. J. Clin. Periodontol..

[30] Teughels W, Van Assche N, Sliepen I, Quirynen M (2006). Effect of material characteristics and/or surface topography on biofilm development. Clin. Oral Implants Res..

[31] Cheon BK (1997). Normal value of mucosal thickness of paranasal sinuses, as seen on brain MRI. J. Korean Radiol. Soc..

