# Supplementary material for: Dental implant as a potential risk factor for maxillary sinus fungus ball
Source: Sci Rep. 2024 Jan 30;14:2483. doi: 10.1038/s41598-024-52661-9 (PMC10827791; doi:10.1038/s41598-024-52661-9)
Supplement: Supplementary file 1 — Supplementary Figure 1. [file 41598_2024_52661_MOESM1_ESM.docx]

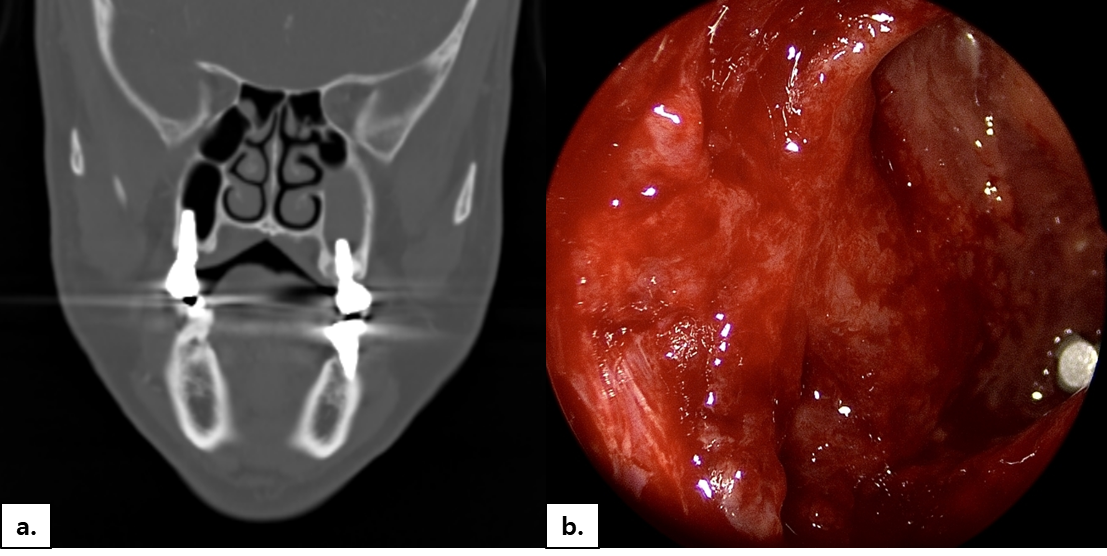


**Supplementary Figure 1.** a. Preoperative CT scan shows the penetrated implant into the maxillary sinus; b. The perforated implant is visible during endoscopic sinus surgery
