# Supplementary material for: Dental implant as a potential risk factor for maxillary sinus fungus ball
Source: Sci Rep. 2024 Jan 30;14:2483. doi: 10.1038/s41598-024-52661-9 (PMC10827791; doi:10.1038/s41598-024-52661-9)
Supplement: Supplementary file 2 — Supplementary Tables. [file 41598_2024_52661_MOESM2_ESM.docx]

| Medical condition | UBS (n = 30) | MFB (n = 30) | *P* value |
| --- | --- | --- | --- |
| Diabetes mellitus, n (%) | 5 | 5 | 0.7611 |
| Liver cirrhosis, n (%) | 1 | 2 |  |
| Hematological malignancy, n (%) | 0 | 0 |  |
| Use of medical immunosuppressants, n (%) | 0 | 1 |  |
| Ongoing chemotherapy, n (%) | 0 | 0 |  |

**Supplementary Table S1.** Underlying medical conditions in age-adjusted patients with unilateral maxillary sinusitis

**Supplementary Table S2.** The number of dental procedures and potential sources of odontogenic sinusitis in each group

|  | **The number of patients**  ***n/total n (%)*** | | | | **The number of teeth**  ***n/total maxillary teeth n (%)*** | | | |
| --- | --- | --- | --- | --- | --- | --- | --- | --- |
|  | UBS | MFB | P-value | OR  (95% CI) | UBS | MFB | P-value | OR  (95% CI) |
| Non-pathological teeth | 9/30 (30) | 11/30 (36.7) 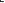 | 0.785 | 1.35 (0.46-3.97) | 81/120 (67.5) | 83/120 (69.1) | 0.889 | 1.08 (0.63-1.86) |
| Root canal treatment | 5/30 (16.7) | 10/30 (33.3) | 0.136 | 2.50 (0.73-8.50) | 9/120 (7.5) | 15/120 (12.5) | 0.197 | 1.76 (0.74-4.20) |
| Dental implant | 1/30 (3.3) | 5/30 (16.7) | 0.085 | 5.80 (0.63-53.01) | 2/120 (1.6) | 9/120 (7.5) | 0.031 | 4.78 (1.01-22.63) |
| Periapical abscess | 4/30 (13.3) | 1/30 (3.3) | 0.161 | 0.22 (0.02-2.14) | 4/120 (3.3) | 2/120 (1.6) | 0.408 | 0.49 (0.09-2.74) |
| Oroantral fistula | 3/30 (10) | 0/30 (0) | 0.076 | ― | 3/120 (2.5) | 0/120 (0) | 0.081 | ― |
| Tooth extraction socket | 9/30 (30) | 6/30 (20) | 0.371 | 0.58 (0.18-1.91) | 21/120 (17.5) | 11/120 (9.2) | 0.058 | 0.48 (0.22-1.04) |

UBS, unilateral bacterial sinusitis; MFB, maxillary sinus fungus ball.
